# Challenges in monitoring the quality of care in multiple sclerosis

**DOI:** 10.1016/j.lanepe.2024.100950

**Published:** 2024-05-25

**Authors:** Uwe Klaus Zettl, Niklas Frahm, Michael Hecker

**Affiliations:** Division of Neuroimmunology, Department of Neurology, Rostock University Medical Centre, Rostock, Germany

We read with interest the publication by Voigt et al. who compiled 24 quality indicators (QIs) and checklists to document and improve the quality of care for people with multiple sclerosis (pwMS).^1^ While useful, we miss a critical evaluation of disease monitoring components.

The developed QIs do not cover diagnostic confirmation. Misdiagnoses continue to be a frequent problem in MS care and typically persist for a long time.^2^ Various disorders can mimic clinical and paraclinical findings of MS. Therefore, careful consideration of alternative diagnoses should be part of the QIs.

The neurological examination includes measuring the patient’s disability level using the Expanded Disability Status Scale (EDSS). Although being a standard tool for decades, the EDSS has several limitations, e.g., low sensitivity for yearly disability progression, high inter-rater variability and under-representation of important symptoms of MS.^3^ The lack of a reliable measure of disability impedes optimal monitoring.

The distinction between disease and intervention monitoring is blurred, as the purpose of querying medications is not explained. We wish to note that polypharmacy is common in pwMS and carries the risk of undesired pharmacological effects due to drug–drug interactions.^4^ Regular medication reviews can reduce inappropriate medication use.

MS specialists and patients also have different quality perceptions. We miss QIs from the patient perspective, such as wait times and overall satisfaction with the care received.^5^

The authors emphasize that the QIs should be regularly adjusted to reflect the current state of research. The aforementioned aspects may be integrated accordingly and the QIs’ added value and practicability further evaluated.

## Contributors

Uwe Klaus Zettl initiated the idea. All authors wrote and edited this letter.

## Declaration of interests

The authors declare no conflict of interest associated with this work.
